# Identification of a *TOP3A* genetic variant as a novel biomarker for sensitivity to doxorubicin

**DOI:** 10.3389/fphar.2026.1724882

**Published:** 2026-05-13

**Authors:** Tana Takacova, Markus Anton Schirmer

**Affiliations:** 1 Asklepios Tumorzentrum Hamburg, Hamburg, Germany; 2 Institute of Clinical Pharmacology, University Medical Center Göttingen, Göttingen, Germany; 3 Clinic of Radiotherapy and Radiation Oncology, University Medical Center Göttingen, Göttingen, Germany

**Keywords:** cytotoxicity, doxorubicin, EC50, lymphoblastoid cell lines, single nucleotide polymorphism, TOP3A, topoisomerase

## Abstract

**Introduction:**

Doxorubicin (DOX), though an effective cytostatic drug, is associated with dose limiting toxicities. Consequently, mandated cumulative-dose restriction may result in compromised tumour control. Improved characterisation of interindividual DOX sensitivity could enable more precise, patient-tailored therapy. We therefore assessed DOX sensitivity in human lymphoblastoid cell lines (LCLs) in relation to common genetic variability in candidate genes encoding five human topoisomerases, putative molecular targets of DOX.

**Methods:**

EC_50_ values for DOX cytotoxicity were determined in 184 LCLs of European ancestry via fluorescence-activated cell sorting. The cohort was split into a training (n = 120) and an independent test (n = 64) set. Comprehensive genotype data were retrieved from the 1,000 Human Genome and the HapMap Project. Across *TOP1*, *TOP2A*, *TOP2B*, *TOP3A*, and *TOP3B*, 1,126 polymorphic sites were identified, with 468 at a minor allele frequency (MAF) ≥ 5%. Associations with EC_50_ in the training set were ranked by *p*-value and evaluated in the test set using a Bonferroni-corrected significance threshold.

**Results:**

In the training set, 12 genetic markers showed associations at *p* < 0.05 with DOX EC_50_ values. One of these, rs113270903 in *TOP3A*, replicated in the test set after multiple testing correction. Variant *T*-allele carriers (*CT* or *TT*) exhibited approximately 30% lower DOX EC_50_ than *CC* homozygotes in both the training and the test sets.

**Conclusion:**

Comprehensive analysis of common diversity in genetic loci coding for human topoisomerases identified rs113270903 in *TOP3A* as a new promising determinant of DOX sensitivity.

## 1 Introduction

Doxorubicin (DOX) is among the oldest cytotoxic chemotherapies, first applied in the 1960s and subsequently adopted across a broad range of malignancies, including breast, lung, gastric, ovarian, thyroid cancer, sarcomas, lymphomas, leukemia, and paediatric malignancies (Carvalho et al., 2009). Beyond antitumour activity, DOX exhibits off-target effects, most importantly dose-dependent cardiotoxicity, which can culminate in irreversible cardiac dysfunction (Carvalho et al., 2009; Jones and Dass, 2022).

This risk is of increasing concern in the context of a globally ageing population, where a substantial proportion of cancer patients present with pre-existing cardiovascular comorbidities. Epidemiological studies estimate that nearly 40% of individuals aged 40–59 already exhibit some form of cardiovascular disease (Yazdanyar and Newman, 2009). Shared risk factors, including obesity, tobacco use, and metabolic syndrome, contribute to the co-occurrence of malignancy and cardiac pathology, compounding the potential for treatment-related complications (Wilcox et al., 2024). Accordingly, there is a pressing clinical need to identify biomarkers that predict both therapeutic efficacy and risk of toxicity to inform patient stratification and guide anthracycline use (Sadee et al., 2011).

Topoisomerase (TOP) II isoforms, particularly *TOP2A* and *TOP2B*, are well-established molecular targets of DOX, and their role in mediating cytotoxicity, intended as well as unintended, has been extensively studied (Pommier et al., 2022; Nitiss, 2009). In contrast, other topoisomerase family members have received limited attention in pharmacogenomic research, despite their critical roles in genome maintenance and DNA topology regulation (Delgado et al., 2018). Among these, *TOP3A* encodes DNA topoisomerase IIIα, a dual-localised enzyme involved in nuclear recombination and mitochondrial genome stability—both compartments relevant to DOX-induced toxicity (Martin et al., 2018; Nicholls et al., 2018; Primiano et al., 2022; Wu and Xiong, 2024).

To address this gap, we conducted a candidate gene analysis for the five human topoisomerases *TOP1*, *TOP2A*, *TOP2B*, *TOP3A,* and *TOP3B*. All proteins encoded by these genes have been shown to participate in cleavage and re-ligation of nuclear DNA during replication and transcription (Pommier et al., 2022). We investigated whether common germline variants in these genes are associated with interindividual variability in DOX sensitivity.

## 2 Methods

### 2.1 Cell lines and culture conditions

Epstein–Barr virus (EBV)-transformed lymphoblastoid cell lines (LCLs) were obtained from the Coriell Institute for Medical Research (Camden, NJ, USA). Cell stocks were cryopreserved in fetal bovine serum (FBS) containing 10% dimethyl sulfoxide (DMSO) and stored at −180 °C in the vapor phase of liquid nitrogen.

For recovery, cryotubes were rapidly thawed at 37 °C and transferred into 50 mL tubes containing 10 mL of ice-cold RPMI-1640 medium (Gibco/Invitrogen, Karlsruhe, Germany), supplemented with 15% heat-inactivated FBS (Thermo Fisher Scientific, Waltham, MA, USA), 2 mM L-glutamine, and 1% penicillin–streptomycin (Thermo Fisher Scientific).

Cell suspensions were centrifuged at 250 *g* for 5 min at 4 °C, and supernatants were discarded. The resulting pellets were resuspended in pre-warmed complete RPMI-1640. Cells were transferred to T25 flasks (Greiner Bio-One GmbH, Kremsmuenster, Austria) and incubated at 37 °C in a humidified atmosphere containing 5% CO_2_. Cultures were passaged two to three times per week based on phenol-red colour change.

No experiments involving live humans or animals were conducted. Analyses were performed on established, anonymised human LCLs and pertinent genotype data openly available. Specific ethics approval for this study was therefore not required.

### 2.2 Flow cytometry

Flow cytometry was used to determine density of viable LCLs prior to chemosensitivity assays and to quantify viability following DOX treatment. Cells were washed twice with phosphate-buffered saline PBS (pH 7.4).

For viability staining, SYTOX™ Green Dead Cell Stain (Thermo Fisher Scientific) diluted at 1:50 corresponding to 1.7 µM was used. CountBright™ Absolute Counting Beads (Thermo Fisher Scientific) were added at 10 µL per 1 mL of cell suspension. Samples were incubated for 15 min at 37 °C, protected from light.

Data were acquired on a BD LSR II SORP fluorescence-activated cell sorting (FACS) machine with excitation lasers used at 405, 488, and 633 nm (Becton Dickinson, Franklin Lakes, NJ, USA). Medium flow rate (∼35 μL/min) was employed, with ≥2,000 bead events and ≥10,000 cell events per sample. Acquisition and initial gating were performed in FACSDiva software, v6.0–8.0 (Becton Dickinson). Cell viability was determined by distinguishing SYTOX-positive (non-viable) from SYTOX-negative (viable) populations based on fluorescence intensity.

Absolute cell density (cells/µL) was calculated using the following formula:

$$\frac{\text{Cells}}{µL}=\frac{\text{cell events}\times \text{No}.\text{ beads added per sample}\times \text{sample dilution}}{\text{bead events}\times \text{sample volume }\left(µL\right)}.$$

### 2.3 Cytotoxicity assay

Chemosensitivity to DOX was assessed in a dose–response viability assay. Doxorubicin hydrochloride (Merck, Darmstadt, Germany) was dissolved in DMSO to 3.45 mM, aliquoted, and stored at −180 °C. Working solutions were freshly diluted in PBS to final concentrations of 0, 5, 10, 20, 40, 80, 160, and 320 nM immediately prior to use.

Prior to DOX exposure, cells were counted by flow cytometry as described above. For the experimental setup, cells were adjusted to a density of 200/µL, and 300 µL of this suspension were employed per reaction in 5-mL tubes compatible with the FACS device. DOX dilutions were added each as 20 µL aliquots to the cell suspensions to achieve the intended final DOX concentrations. For drug-free controls, PBS was used instead. All conditions were performed in duplicate. Suspensions were incubated upright at 37 °C in 5% CO_2_ for 48 h. Post-treatment viability was assessed by FACS.

Chemosensitivity to DOX was evaluated by calculating the half-maximal effective concentration (EC_50_) of DOX from an eight-point dose-response curve. The curve included DOX concentrations ranging from 0 to 320 nM, each ascertained in duplicate, and fitted with the four-parametric Hill logistic model. Curve residuals were assessed as *r* ^*2*^-values between the observed data and the model fit. Only LCLs with *r* ^*2*^ ≥ 0.90 were considered for further analysis (median *r* ^*2*^ = 0.99 for the used 184 LCLs indicating an excellent goodness-of-fit). For included as well as excluded LCLs, please refer to Supplementary Table S1.

### 2.4 Candidate genes and genotype data

The five human topoisomerase genes *TOP1*, *TOP2A*, *TOP2B*, *TOP3A*, and *TOP3B* were selected as candidate genes for DOX sensitivity. Expression of these genes in LCLs was confirmed using normalised transcriptomic data from BioGPS (http://biogps.org) (Scripps Research Institute, 2007). Germline genotypes were retrieved from the International HapMap Project and the 1000 Genomes Project (International HapMap Consortium, 2007; Auton et al., 2015). These data concerned the genomic regions of the considered five topoisomerase genes, each including 5 kb flanking regions upstream and downstream to cover potential proximal cis-regulatory elements. Genetic markers were filtered for a minor allele frequency (MAF) ≥ 5%. Using HaploView software (version 4.2) (Barrett et al., 2005), pairwise linkage disequilibrium (LD) was visualised and the therein implemented tagger algorithm was employed to define tagging markers covering others in high LD with a threshold of *r* ^*2*^ ≥ 0.80 applied. All markers were checked for accordance with the Hardy-Weinberg equilibrium (HWE). Significant deviations are usually assumed if *p*-values for HWE (*p*_*HWE*_) are < 0.001.

### 2.5 Statistical analysis

Assuming a mean difference of 5 nM and a standard deviation of 10 nM in EC_50_ for doxorubicin would result in a total sample size of 126 LCLs when comparing two equally sized groups with a type I error of 5% and a test power of 80%. As genotype groups typically differ in size due to varying allele frequencies, the sample size required under the same assumptions may increase accordingly to reliably detect a true association.

A total of 200 unrelated LCLs of Caucasian ethnicity were initially considered for analyses. Five were excluded due to poor growth and one due to non-availability of any genotype data. EC_50_ values could not be derived in ten samples due to insufficient Hill model convergence. Thus, 184 LCLs were available for genotype-phenotype analysis. Of these LCLs, 101 derived from donors of Utah residents with Northern and Western European ancestry (CEU), and 83 of British (GBR) descent. The included individual LCL identifiers with the EC_50_, values for DOX as well as those excluded from analysis are provided in Supplementary Table S1 in Supplementary Material.

Associations between EC_50_ values and genotypes were tested using the Jonckheere–Terpstra trend test, which is non-parametric and rank-based. This test accounts for gene-dose effects across ordered genotype groups (wild-type homozygotes, heterozygotes, and variant homozygotes). In case of only two genotype configurations of an independent variable, the Mann-Whitney U test was employed. The 184 LCLs were split into a training (n = 120) and a test (n = 64) set. In the training set, genetic markers were ranked by ascending *p*-values. This order was used for assessments in the test set applying a step-down multiple testing correction according to the Bonferroni adjustment (e.g., *p* < 0.05 for the marker with the strongest association in the training set, *p* < 0.025 for the second strongest, etc.). We chose this method because our primary goal was to keep the type I error rate low by strictly controlling the family-wise error rate. This stepwise procedure strongly reduces the type I error rate compared to unadjusted *p*-values and is, in this regard, more conservative than alternative approaches such as Holm-Bonferroni or Benjamini–Hochberg. At the same time, it is less conservative than conventional Bonferroni, thereby reducing the risk of type II errors and thus enhances the probability to detect potentially relevant associations. Consequently, we considered the stepwise Bonferroni correction to provide an adequate balance between false positive and false negative findings in our setting of a limited number of pre-specified comparisons.

In addition to the univariable tests, we performed models in which the impact of a specific genetic marker on the dependent EC_50_ variable was adjusted for population substructure (the two Caucasian populations) or training versus test set. Therefore, full factorial general linear models with type III sum of squares were evaluated. The EC_50_ of doxorubicin was modelled as the dependent variable, whereas an identified genetic marker of interest in conjunction with population (CEU versus GBR) and study part (training versus test set) was considered as independent fixed factors. Logarithmic transformation of the original EC_50_ data, which were substantially skewed to the right, resulted in acceptable homoscedasticity (i.e., equality of error variances) and linearity as assessed by Levene’s test, variance ratio and visual inspection of residual plots.

All analyses were carried out using SPSS Statistics, version 27 (IBM Corp., Armonk, NY, USA).

## 3 Results

### 3.1 Validation of EC_50_ measurement reproducibility

**FIGURE 1:**
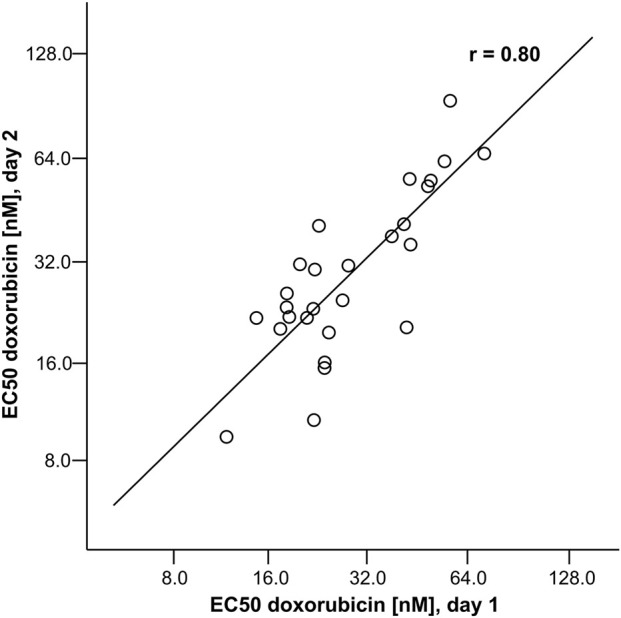
Reproducibility of EC_50_ assessment for DOX. The data presented refer to 27 LCLs (13 of the training and 14 of the test set), for which EC_50_ values were ascertained two times independently at different days. For reasons of compatibility with normal distribution data were log-transformed with basis 2. This transformation resulted in *p*-values ≥0.2 for both day 1 and day 2 according to Shapiro-Wilk test indicating no statistically significant deviation from normal distribution. A regression line with the Pearson correlation coefficient *r* is drawn.

For genotype-phenotype analyses, a reproducible phenotype is indispensable. Thus, EC_50_ values were independently determined for 27 LCLs on two occasions. Pearson’s correlation was *r* = 0.80 (*p* = 6 × 10^−7^), supporting assay reproducibility (Figure 1).

### 3.2 Distribution of DOX chemosensitivity

**FIGURE 2:**
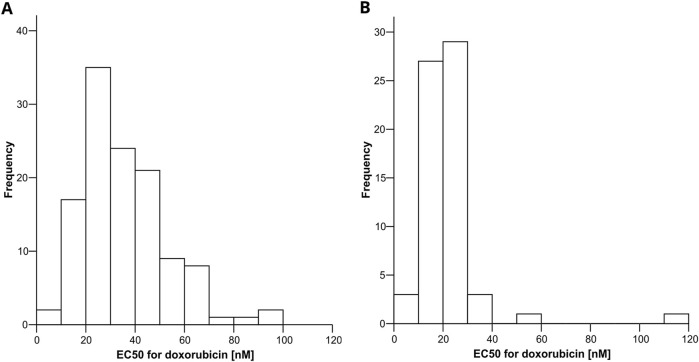
Distribution of EC_50_ for DOX in the training (panel **(A)**) and test **(B)** set.

In the full cohort of 184 LCLs, the median EC_50_ value for DOX was 25.7 nM. The interquartile range (IQR) was 19.7–39.1 nM. The lowest EC_50_ was 5.8 nM in the most sensitive LCL, and the highest was 112.1 nM in the most resistant LCL. The EC_50_ distributions separated for the training and test set are displayed in Figure 2.

### 3.3 Distribution of topoisomerase gene polymorphisms

Based on 1,000 Genomes CEU/GBR data, 1,126 polymorphic sites were annotated within the five topoisomerase genes including ±5 kb flanking regions (Table 1). This corresponds to a marker density between 2.6 (*TOP2A*) and 4.9 (*TOP3A*) per 1 kb. After filtering for MAF ≥ 5%, 468 variants (42.6%) remained and were analysed for association with EC_50_ in the training set.

**TABLE 1:** Polymorphic sites in genes encoding human topoisomerases. Data are based on the 184 LCLs (training and test set) employed for the EC_50_ assessment of DOX.

Gene name	All polymorphic sites		Sites with MAF ≥ 5%		Sites with MAF ≥ 5% and LD at r^2^ < 0.80	
	Total (n)	per 1 kb	Total (n)	per 1 kb	Total (n)	per 1 kb
TOP1	337	3.24	128	1.23	23	0.22
TOP2A	102	2.61	17	0.43	8	0.20
TOP2B	262	3.43	116	1.52	18	0.24
TOP3A	264	4.9	115	2.1	15	0.28
TOP3B	161	4.5	92	2.6	20	0.57
Sum	1,126	​	468	​	84	​

Only one single marker (*TOP1* rs6016504) failed the HWE criterion and was therefore excluded from further analysis. All other genetic markers featured *p*_*HWE*_ ≥ 0.05 indicating high fidelity in the genotype data.

### 3.4 Associations of topoisomerase gene polymorphisms with DOX EC_50_

We observed 468 variant genetic sites with a MAF ≥ 5% in the five human topoisomerase genes. Upon filtering for high genetic linkage disequilibrium (LD), i.e., only variants with a pairwise LD at *r* ^*2*^ < 0.80 with any other variant were considered as independent (Table 1, right column). This resulted in 84 genetic polymorphisms evaluated in the training set. Out of these, 12 demonstrated nominally significant associations with the EC_50_ of DOX (*p* < 0.05; Table 2). These associations were ranked by ascending *p*-values to guide stepwise multiplicity correction in the test set, i.e., no correction was applied to the top-ranked marker (*p* = 0.05), while increasingly stringent thresholds were used for subsequent ones (e.g., *p* = 0.05/2 for the second, 0.05/3 for the third, etc.).

**TABLE 2:** Association of genetic markers in topoisomerase genes with EC_50_ of DOX as assessed by the Jonckheere-Terpstra trend test. Markers at *p* < 0.05 in the training are listed in increasing order according to the *p* value. In case of high linkage disequilibrium (i.e., *r* ^*2*^ ≥ 0.80) between two or more markers one representative is shown. The denoted *p* values in this list are not corrected for multiple testing.

Gene	Rs number	Training set	Test set
*TOP1*	rs6016505	0.005	0.761
*TOP2A*	rs190471737	0.009	0.481
*TOP3A*	rs7221807	0.012	0.676
*TOP3B*	rs9610794	0.013	0.074
*TOP2A*	rs13695	0.013	0.850
*TOP2A*	rs2715555	0.014	0.469
*TOP3A*	rs113270903	0.028	0.0007
*TOP1*	rs11697710	0.035	0.687
*TOP3B*	rs2283797	0.044	0.312
*TOP3A*	rs8076253	0.044	0.718
*TOP3B*	rs75104161	0.047	0.993
*TOP3A*	rs77697889	0.048	0.282

**FIGURE 3:**
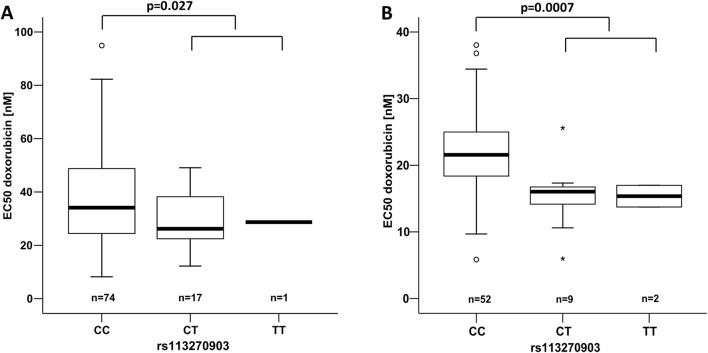
EC_50_ for DOX in dependence on *TOP3A* rs113270903. The left panel **(A)** shows the training, the right **(B)** the test set. The denoted *p*-values refer to the Mann-Whitney U test comparing the major allele homozygous genotype configuration with the combined heterozygous and the homozygous minor allele state. The numbers differ from the total of 184 LCLs due to missing genotypes in the accessed databases.

Of the 12 candidate markers with *p* < 0.05 in the training set, only rs113270903, located in intron 17 of *TOP3A*, maintained statistical significance in the test set (*p* = 0.0007, i.e., *p*_*limit*_ = 0.05/7 = 0.0071 upon multiplicity testing correction as placed seventh in the training set). This variant, with a minor allele frequency of 9.5% in individuals of European ancestry, was consistently associated with increased DOX sensitivity, as evidenced by approximately 30% lower EC_50_ values in both the training and test sets. Genotype-stratified EC_50_ distributions for rs113270903 are shown in Figure 3. In the combined dataset of the training and test sets, the mean difference in EC50 between *CC* and *CT* + *TT* genotypes was 8.74 nM, with a 95% confidence interval of 2.00–15.47 nM.

As the overall distributions of EC_50_ values differ between the training and test sets, we performed an analysis on the entire sample set, adjusting the effect of *TOP3A* rs113270903 for study part (training versus test). The two populations, from which the LCLs were derived (CEU versus GBR), were considered as a further potential confounder. Accordingly, we employed a full factorial univariate general linear model with EC_50_ values as the dependent variable with *TOP3A* rs113270903, study part, and population as independent fixed factors. This analysis confirmed *TOP3A* rs113270903 (*p* = 8*10^–5^) and study part (*p* = 2*10^–8^) as statistically significant, whilst population was not (*p* = 0.273). All four interaction terms in this model exhibited *p*-values > 0.05, indicating they were not relevant. Overall, this general linear model corroborates the impact of *TOP3A* rs113270903 on cellular sensitivity to doxorubicin.

Another genetic marker, *TOP3B* rs9610794, with a MAF of 15% in the sample set investigated, showed a trend-like association in the test set (*p* = 0.074; Table 2) without reaching the pre-specified level of statistical significance. Notably, the minor allele of this variant was associated with an increased EC_50_ of doxorubicin in both the training and test sets.

Full lists of the polymorphic markers, those with a MAF ≥ 5%, and tagging markers selected for association testing are provided in the Supplementary Excel file S2, with separate sheets for each of the five considered genes.

## 4 Discussion

We identified a statistically significant association between an intronic single nucleotide polymorphism (SNP) in *TOP3A* and *in vitro* DOX chemosensitivity. Carriers of the rs113270903 allele demonstrated ∼30% lower EC_50_ values compared to non-carriers across both training and test sets, indicating enhanced chemosensitivity. This replicated finding in two independent cohorts supports the robustness of the *TOP3A* rs113270903 association. No associations at the pre-specified level of statistical significance were observed for other markers in *TOP3A*, nor for any in *TOP1*, *TOP2A*, *TOP2B*, or *TOP3B* after correction for multiple testing. However, the trend-level association of *TOP3B* rs9610794 in the test set, together with a minor allele effect in the same direction as in the training set, may render this marker an interesting candidate for future investigations.

In addition to identifying *TOP3A* rs113270903 as a novel marker of DOX sensitivity, our results suggest a previously unrecognized interaction between DOX and *TOP3A*, which, to our best knowledge, has not yet been described in literature. Thus, our study also suggests a new drug-protein relationship that warrants further mechanistic and clinical evaluation.

A single *TOP3A* transcript gives rise to two protein (Topo IIIα) isoforms directed to two different cellular compartments via alternative translation initiation. The upstream start site produces a mitochondrial isoform that resolves hemicatenanes near the heavy-strand origin of mitochondrial DNA (mtDNA) replication, enabling decatenation, nucleoid segregation, and maintenance of mtDNA copy number. Translation of the downstream portion of the *TOP3A* transcript part generates the nuclear isoform, which dissolves double Holliday junctions to preserve genomic integrity during recombination (Erdinc et al., 2023). Topo IIIα acts as a type IA topoisomerase, relieving DNA topological stress via adenosine triphosphate (ATP)-independent single-strand passage (Wang et al., 2025).

Apart from cardiolipin-associated accumulation in cardiomyocytes, DOX has been reported to localize to mitochondria in non-cardiac cell models, including HeLa and MCF-7 cells (Bellance et al., 2020; Xu et al., 2014). DOX exposure has been reported to induce mitochondrial genome topological stress, characterized by increased mtDNA supercoiling/catenation and concomitant mtDNA nucleoid aggregation (Lei et al., 2023). In this context, mitochondrial Topo IIIα is uniquely required for hemicatenane resolution in this compartment, as no other mitochondrial topoisomerase can catalyse this ATP-independent strand passage: Mitochondrial topoisomerase I (Topo Imt) only relaxes supercoils without strand passage, while Topo IIβ forms covalent 5′-phosphotyrosyl cleavage complexes that are inefficiently resolved in mitochondria, resulting in persistent protein-linked double-strand breaks that can impede replication and transcription (Wang et al., 2025). Consistent with a central role for Topo IIIα in mtDNA metabolism, *TOP3A* depletion has been shown to cause replication fork stalling, accompanied by increased mtDNA catenation and a reduction in mtDNA copy number. Conversely, although Topo IIIα overexpression can accelerate clearance of replication termination intermediates and facilitate replication completion, excessive Topo IIIα activity may increase local mtDNA fragility by promoting strand cleavage within single stranded DNA-rich structures (which occur temporarily during replication) near the heavy-strand origin (Hangas et al., 2022). Together, these observations indicate that both deficiency and dysregulated excess of Topo IIIα perturb mtDNA maintenance and transcription.

Biallelic pathogenic variants in the coding region of *TOP3A* were reported to produce distinct clinical phenotypes consistent with the enzyme’s dual nuclear–mitochondrial localization. Loss of nuclear *TOP3A* function results in a Bloom-like disorder characterised by chromosomal instability due to excessive sister chromatid hyperrecombination, growth restriction, immunodeficiency, and cancer predisposition (Martin et al., 2018). In contrast, mitochondrial *TOP3A* dysfunction manifests as an adult-onset mtDNA-maintenance disorder with multiple deletions or depletion, implicating progressive ophthalmoplegia, myopathy, neuropathy, hearing loss, and cardiac conduction defects (Nicholls et al., 2018; Primiano et al., 2022).

The location of rs113270903 in the penultimate intron of *TOP3A* suggests a noncoding regulatory mechanism rather than a coding effect, potentially involving altered expression, splicing, or a shift in the balance between mitochondrial and nuclear isoforms (Sadee et al., 2011; Erdinc et al., 2023). Given the distinct, non-redundant roles of these two Topo IIIα isoforms, even subtle perturbations in regulation could have compartment-specific consequences. A preferential reduction of the mitochondrial isoform would impair mtDNA decatenation and nucleoid segregation, lowering the threshold for DOX-induced mitochondrial toxicity (Nicholls et al., 2018; Wu and Xiong, 2024). Conversely, an effect concerning the cell nucleus could influence the resolution of potentially harmful recombination intermediates generated by Topo IIα and IIβ, the translational products of *TOP2A* and *TOP2B* (Wu and Xiong, 2024).

Alternative lengthening of telomeres (ALT) has been proposed as a mechanism supporting replicative immortality in approximately 10%–15% of cancers (Khandagale et al., 2024). *TOP3A* has recently been reported to be implicated as a key effector in this pathway, functioning to resolve recombination intermediates and preserve telomere integrity (Khandagale et al., 2024; Khandagale et al., 2025). Additional studies have demonstrated elevated telomere-localized replication stress as a characteristic feature of ALT-positive tumours (Dubois et al., 2025). Collectively, these findings suggest that *TOP3A* may represent a promising therapeutic target for further investigation.

Previous research on topoisomerase-mediated DOX sensitivity has focused primarily on the *TOP2* subtypes (Pommier et al., 2022). While *TOP2A* amplification and overexpression have been associated with increased drug response in some tumor types, findings remain inconsistent (Arriola et al., 2007; Di Leo et al., 2011). Topo IIβ, by contrast, is more relevant to DOX-induced off-target cardiotoxicity, where trapping Topo IIβ–DNA cleavage complexes in cardiomyocytes impairs mitochondrial transcription and bioenergetics (Wang et al., 2025).

Limitations of our study include the fact that *in vitro* pharmacodynamic measurements in a cell model do not account for host pharmacokinetics, metabolism, or tissue-specific gene regulation. This affects the generalizability and transferability of our findings to complex organisms and clinical settings. Our assessments were restricted to individuals of Caucasian ancestry, thus may not be directly transferable to other populations due to inter-ethnic differences in the genetic and epigenetic background affecting, *inter alia*, gene expression regulation. Thus, pending clinical validation, the finding for *TOP3A* rs113270903 as for now is limited to the applied *in vitro* model of LCLs. However, LCLs might fairly well reflect fast-dividing cells and can therefore be regarded as a suitable surrogate for leukemic malignancies in which doxorubicin is often used. It remains to be demonstrated whether the reported genotype-phenotype association is actually relevant for target cells of doxorubicin therapy. The use of immortalized B cells may not capture lineage-dependent characteristics, such as differences in the expression of transporter and DNA repair proteins, which vary among different types of human cancer. Accordingly, rs113270903 should be considered a candidate marker identified in an *in vitro* model pending clinical validation. Another limitation is the size of the evaluated LCL cohorts, with reduced power to detect potential effects, especially from low-frequency genetic variants. A genome wide analysis is not appropriate with this sample size.

Besides replication in cell line from other ancestries, future work should include assessment of rs113270903 in doxorubicin-treated patients. Association analyses should be undertaken with respect to relevant endpoints such as adverse events (e.g., cardiotoxicity) and treatment outcomes (e.g., response and survival).

This study has several notable strengths. The fast-dividing LCLs may, to some extent, mimic highly proliferative haematologic or solid malignancies. As immortalized B-lymphocytes, these genetically characterised cells provide a contextually relevant model for assessing genetic determinants of doxorubicin response, given the drug´s established role in leukaemia treatment (Mamun et al., 2025). Cytotoxicity constitutes a complex endpoint influenced by numerous factors. Homogeneity of experimental setup enhances sensitivity to germline variant effects, which was ensured through our protocol. In this line, duplicate assays performed on two independent occasions in 27 LCLs (13 training set; 14 test set) demonstrated high intra-cell line reproducibility.

## 5 Conclusion

A comprehensive association analysis of 468 common germline variants in *TOP1*, *TOP2A*, *TOP2B*, *TOP3A*, and *TOP3B* in 184 LCLs identified a reproducible and statistically significant association between the intronic variant rs113270903 in *TOP3A* and ∼30% lower doxorubicin EC_50_. Given the narrow therapeutic window of doxorubicin, this finding may inform genotype-guided treatment stratification. To establish clinical relevance, further studies are required. These include confirmation in patient cohorts and mechanistic investigations related to the observed effect of the *TOP3A* rs113270903 genetic marker.

## Data Availability

The datasets presented in this study can be found in online repositories. The names of the repository/repositories and accession number(s) can be found in the article/Supplementary Material.
