# Supplementary material for: Identification of a TOP3A genetic variant as a novel biomarker for sensitivity to doxorubicin
Source: Front Pharmacol. 2026 May 13;17:1724882. doi: 10.3389/fphar.2026.1724882 (PMC13213166; doi:10.3389/fphar.2026.1724882)
Supplement: Supplementary file 2 [file Table1.docx]

**Table S1. List of sample cell lines initially included in the study and their identifiers.** A total of 200 LCLs were selected for evaluation of doxorubicin sensitivity, determined as EC50 values for cytotoxicity. EC₅₀ data could not be obtained for 16 LCLs: five exhibited poor growth (GM12045, GM12340, GM12342, GM12413, GM12716), EC₅₀ estimation failed in ten (GM11829, GM12046, GM12341, GM12383, GM12815, HG00125, HG00135, HG00136, HG00142, HG00242) - primarily due to low event counts during flow cytometric analysis. When genotypes were accessed, one cell line (GM12414) lacked data. The remaining 184 LCLs with valid EC₅₀ data were randomly allocated into a training set (n = 120) and a test set (n = 64) in a 2:1 ratio.

| **Coriell number** | **Population code** | **Included/**  **not included (reason)** | **Training/**  **test set** | **Final EC_50_ for doxorubicin [nM]** |
| --- | --- | --- | --- | --- |
| GM11829 | CEU | no (EC_50_ failed) | n.a. | n.a. |
| GM12045 | CEU | no (poor growth) | n.a. | n.a. |
| GM12046 | CEU | no (EC_50_ failed) | n.a. | n.a. |
| GM12340 | CEU | no (poor growth) | n.a. | n.a. |
| GM12341 | CEU | no (EC_50_ failed) | n.a. | n.a. |
| GM12342 | CEU | no (poor growth) | n.a. | n.a. |
| GM12383 | CEU | no (EC_50_ failed) | n.a. | n.a. |
| GM12413 | CEU | no (poor growth) | n.a. | n.a. |
| GM12414 | CEU | no (missing genotypes) | n.a. | n.a. |
| GM12716 | CEU | no (poor growth) | n.a. | n.a. |
| GM12815 | CEU | no (EC_50_ failed) | n.a. | n.a. |
| HG00125 | GBR | no (EC_50_ failed) | n.a. | n.a. |
| HG00135 | GBR | no (EC_50_ failed) | n.a. | n.a. |
| HG00136 | GBR | no (EC_50_ failed) | n.a. | n.a. |
| HG00142 | GBR | no (EC_50_ failed) | n.a. | n.a. |
| HG00242 | GBR | no (EC_50_ failed) | n.a. | n.a. |
| GM06984 | CEU | yes | 1 | 40.5 |
| GM06985 | CEU | yes | 1 | 23.1 |
| GM06986 | CEU | yes | 1 | 31.3 |
| GM06989 | CEU | yes | 1 | 41.4 |
| GM06993 | CEU | yes | 1 | 26.0 |
| GM06994 | CEU | yes | 1 | 17.7 |
| GM07000 | CEU | yes | 1 | 22.2 |
| GM07022 | CEU | yes | 1 | 39.2 |
| GM07034 | CEU | yes | 1 | 50.7 |
| GM07037 | CEU | yes | 1 | 41.3 |
| GM07051 | CEU | yes | 1 | 40.3 |
| GM07055 | CEU | yes | 1 | 40.9 |
| GM07056 | CEU | yes | 1 | 29.4 |
| GM07345 | CEU | yes | 1 | 60.4 |
| GM07346 | CEU | yes | 1 | 40.9 |
| GM07347 | CEU | yes | 1 | 23.4 |
| GM07357 | CEU | yes | 1 | 45.7 |
| GM11830 | CEU | yes | 1 | 58.7 |
| GM11831 | CEU | yes | 1 | 27.8 |
| GM11832 | CEU | yes | 1 | 18.8 |
| GM11839 | CEU | yes | 1 | 27.2 |
| GM11840 | CEU | yes | 1 | 41.1 |
| GM11843 | CEU | yes | 1 | 23.2 |
| GM11881 | CEU | yes | 1 | 23.9 |
| GM11882 | CEU | yes | 1 | 38.9 |
| GM11892 | CEU | yes | 1 | 56.5 |
| GM11992 | CEU | yes | 1 | 82.3 |
| GM11993 | CEU | yes | 1 | 22.4 |
| GM11994 | CEU | yes | 1 | 40.9 |
| GM11995 | CEU | yes | 1 | 29.1 |
| GM12003 | CEU | yes | 1 | 26.3 |
| GM12004 | CEU | yes | 1 | 27.0 |
| GM12006 | CEU | yes | 1 | 33.6 |
| GM12043 | CEU | yes | 1 | 29.1 |
| GM12044 | CEU | yes | 1 | 16.2 |
| GM12056 | CEU | yes | 1 | 29.3 |
| GM12057 | CEU | yes | 1 | 64.7 |
| GM12144 | CEU | yes | 1 | 22.2 |
| GM12145 | CEU | yes | 1 | 32.1 |
| GM12146 | CEU | yes | 1 | 26.0 |
| GM12154 | CEU | yes | 1 | 19.7 |
| GM12155 | CEU | yes | 1 | 43.7 |
| GM12156 | CEU | yes | 1 | 90.1 |
| GM12234 | CEU | yes | 1 | 17.6 |
| GM12239 | CEU | yes | 1 | 50.4 |
| GM12248 | CEU | yes | 1 | 32.6 |
| GM12249 | CEU | yes | 1 | 8.2 |
| GM12275 | CEU | yes | 1 | 55.0 |
| GM12282 | CEU | yes | 1 | 60.2 |
| GM12283 | CEU | yes | 1 | 31.6 |
| GM12286 | CEU | yes | 1 | 24.3 |
| GM12347 | CEU | yes | 1 | 18.0 |
| GM12348 | CEU | yes | 1 | 36.8 |
| GM12399 | CEU | yes | 1 | 36.6 |
| GM12717 | CEU | yes | 1 | 65.5 |
| GM12748 | CEU | yes | 1 | 68.9 |
| GM12750 | CEU | yes | 1 | 16.7 |
| GM12751 | CEU | yes | 1 | 24.4 |
| GM12760 | CEU | yes | 1 | 19.4 |
| GM12761 | CEU | yes | 1 | 26.2 |
| GM12762 | CEU | yes | 1 | 24.2 |
| GM12763 | CEU | yes | 1 | 30.3 |
| GM12775 | CEU | yes | 1 | 29.5 |
| GM12776 | CEU | yes | 1 | 18.9 |
| GM12777 | CEU | yes | 1 | 52.5 |
| GM12778 | CEU | yes | 1 | 32.3 |
| GM12812 | CEU | yes | 1 | 30.6 |
| GM12813 | CEU | yes | 1 | 8.9 |
| GM12814 | CEU | yes | 1 | 26.7 |
| GM12827 | CEU | yes | 1 | 19.1 |
| GM12828 | CEU | yes | 1 | 31.9 |
| GM12829 | CEU | yes | 1 | 42.3 |
| GM12830 | CEU | yes | 1 | 75.3 |
| GM12842 | CEU | yes | 1 | 65.4 |
| GM12843 | CEU | yes | 1 | 39.7 |
| GM12872 | CEU | yes | 1 | 13.5 |
| GM12873 | CEU | yes | 1 | 20.8 |
| GM12874 | CEU | yes | 1 | 45.8 |
| GM12875 | CEU | yes | 1 | 18.4 |
| GM12889 | CEU | yes | 1 | 39.6 |
| GM12890 | CEU | yes | 1 | 94.9 |
| GM12891 | CEU | yes | 1 | 13.0 |
| GM12892 | CEU | yes | 1 | 21.1 |
| HG00099 | GBR | yes | 1 | 65.6 |
| HG00100 | GBR | yes | 1 | 24.1 |
| HG00101 | GBR | yes | 1 | 22.5 |
| HG00102 | GBR | yes | 1 | 41.0 |
| HG00103 | GBR | yes | 1 | 53.9 |
| HG00104 | GBR | yes | 1 | 33.8 |
| HG00106 | GBR | yes | 1 | 36.7 |
| HG00108 | GBR | yes | 1 | 31.0 |
| HG00113 | GBR | yes | 1 | 53.1 |
| HG00114 | GBR | yes | 1 | 31.2 |
| HG00116 | GBR | yes | 1 | 55.8 |
| HG00117 | GBR | yes | 1 | 24.3 |
| HG00120 | GBR | yes | 1 | 30.5 |
| HG00121 | GBR | yes | 1 | 24.1 |
| HG00122 | GBR | yes | 1 | 28.7 |
| HG00123 | GBR | yes | 1 | 32.8 |
| HG00124 | GBR | yes | 1 | 17.4 |
| HG00126 | GBR | yes | 1 | 47.3 |
| HG00127 | GBR | yes | 1 | 49.7 |
| HG00129 | GBR | yes | 1 | 17.6 |
| HG00130 | GBR | yes | 1 | 49.1 |
| HG00131 | GBR | yes | 1 | 48.8 |
| HG00133 | GBR | yes | 1 | 21.1 |
| HG00134 | GBR | yes | 1 | 21.5 |
| HG00137 | GBR | yes | 1 | 36.9 |
| HG00138 | GBR | yes | 1 | 67.1 |
| HG00139 | GBR | yes | 1 | 48.9 |
| HG00140 | GBR | yes | 1 | 43.0 |
| HG00141 | GBR | yes | 1 | 18.8 |
| HG00143 | GBR | yes | 1 | 26.4 |
| HG00146 | GBR | yes | 1 | 23.2 |
| HG00148 | GBR | yes | 1 | 34.4 |
| HG00149 | GBR | yes | 1 | 40.7 |
| HG00150 | GBR | yes | 1 | 12.2 |
| HG00151 | GBR | yes | 1 | 27.7 |
| HG00152 | GBR | yes | 1 | 38.2 |
| HG00247 | GBR | yes | 1 | 40.5 |
| GM11893 | CEU | yes | 2 | 13.0 |
| GM11894 | CEU | yes | 2 | 6.0 |
| GM11918 | CEU | yes | 2 | 20.2 |
| GM11919 | CEU | yes | 2 | 5.8 |
| GM11920 | CEU | yes | 2 | 23.7 |
| GM11930 | CEU | yes | 2 | 18.8 |
| GM11931 | CEU | yes | 2 | 25.4 |
| GM11932 | CEU | yes | 2 | 19.6 |
| GM11933 | CEU | yes | 2 | 17.9 |
| GM12058 | CEU | yes | 2 | 112.1 |
| GM12272 | CEU | yes | 2 | 26.5 |
| GM12273 | CEU | yes | 2 | 16.3 |
| GM12287 | CEU | yes | 2 | 13.7 |
| GM12400 | CEU | yes | 2 | 28.8 |
| GM12489 | CEU | yes | 2 | 25.2 |
| GM12546 | CEU | yes | 2 | 51.2 |
| GM12718 | CEU | yes | 2 | 24.8 |
| GM12749 | CEU | yes | 2 | 21.5 |
| HG00096 | GBR | yes | 2 | 16.7 |
| HG00097 | GBR | yes | 2 | 21.6 |
| HG00109 | GBR | yes | 2 | 25.6 |
| HG00110 | GBR | yes | 2 | 22.2 |
| HG00111 | GBR | yes | 2 | 18.2 |
| HG00112 | GBR | yes | 2 | 17.3 |
| HG00118 | GBR | yes | 2 | 34.4 |
| HG00119 | GBR | yes | 2 | 21.2 |
| HG00154 | GBR | yes | 2 | 22.6 |
| HG00155 | GBR | yes | 2 | 15.2 |
| HG00156 | GBR | yes | 2 | 10.6 |
| HG00158 | GBR | yes | 2 | 19.7 |
| HG00159 | GBR | yes | 2 | 12.8 |
| HG00160 | GBR | yes | 2 | 16.1 |
| HG00231 | GBR | yes | 2 | 20.7 |
| HG00232 | GBR | yes | 2 | 21.9 |
| HG00233 | GBR | yes | 2 | 27.2 |
| HG00234 | GBR | yes | 2 | 17.6 |
| HG00235 | GBR | yes | 2 | 19.9 |
| HG00236 | GBR | yes | 2 | 38.0 |
| HG00237 | GBR | yes | 2 | 36.8 |
| HG00238 | GBR | yes | 2 | 16.0 |
| HG00239 | GBR | yes | 2 | 22.0 |
| HG00240 | GBR | yes | 2 | 18.9 |
| HG00243 | GBR | yes | 2 | 22.0 |
| HG00244 | GBR | yes | 2 | 22.6 |
| HG00245 | GBR | yes | 2 | 18.5 |
| HG00246 | GBR | yes | 2 | 9.7 |
| HG00249 | GBR | yes | 2 | 15.2 |
| HG00250 | GBR | yes | 2 | 23.8 |
| HG00251 | GBR | yes | 2 | 17.0 |
| HG00252 | GBR | yes | 2 | 14.2 |
| HG00253 | GBR | yes | 2 | 20.8 |
| HG00254 | GBR | yes | 2 | 20.0 |
| HG00255 | GBR | yes | 2 | 22.7 |
| HG00256 | GBR | yes | 2 | 25.7 |
| HG00257 | GBR | yes | 2 | 17.3 |
| HG00258 | GBR | yes | 2 | 18.3 |
| HG00259 | GBR | yes | 2 | 20.9 |
| HG00260 | GBR | yes | 2 | 22.2 |
| HG00261 | GBR | yes | 2 | 25.9 |
| HG00262 | GBR | yes | 2 | 22.1 |
| HG00263 | GBR | yes | 2 | 20.3 |
| HG00264 | GBR | yes | 2 | 25.8 |
| HG00265 | GBR | yes | 2 | 16.4 |
| HG01334 | GBR | yes | 2 | 10.7 |

n.a. = not applicable
